# Impacts of nitrogen fertilization and planting date on the physiology and yield of purple sweet potato at the extreme Northern edge of cultivation

**DOI:** 10.1371/journal.pone.0318531

**Published:** 2025-03-26

**Authors:** Ivar Zekker, Astrid Kännaste, Viacheslav Eremeev, Kaia Kask, Pille Meinson, Helina Nassar, Erkki Mäeorg, Eve Runno-Paurson, Ülo Niinemets

**Affiliations:** 1 Chair of Crop Science and Plant Biology, Institute of Agricultural and Environmental Sciences, Estonian University of Life Sciences, Tartu, Estonia; 2 Estonian Academy of Sciences, Tallinn, Estonia; United Arab Emirates University, UNITED ARAB EMIRATES

## Abstract

Global warming causes plant stress and reduces crop productivity. Cultivation of the warmer region crop sweet potato (*Ipomoea batatas* (L.) Lam) in Northern regions can be an opportunity to benefit from climate warming, but there is little information of how growing season length interacts with agricultural practices such as nitrogen (N) fertilization. We studied the photosynthetic characteristics, biomass accumulation, carbon (C) and N contents of plant organs of the cultivar ‘Purple Bud’ in relation to the planting date (the 2nd of May, 10th of May, 20th of May, 30th of May and 10th of June) and N fertilization (kg ha^-1^; N0, N50, N100 and N150). Nitrogen content of leaves (*N*_L_) and tubers (*N*_T_) increased with N application dose and was moderately affected by planting time. Despite the fertilization-dependent increase of leaf N content, photosynthesis rate (*A*) was unaffected or somewhat reduced by N fertilization. This reflected reductions in stomatal conductance (*g*_s_) and ratio of intercellular CO_2_ to ambient CO_2_ (*C*_i_/*C*_a_), suggesting that enhanced N availability and concomitant increase in whole plant area resulted in reduced plant water availability. The highest values of leaf C/N ratio, tuber to root mass ratio and dry weight content of roots (*DW*_R_) were found in N0 plants and the ones planted on the 10th of May and 20th of May. Our results collectively demonstrate that the growth and productivity of sweet potato is strongly dependent on the length of the growing season, and can be further constrained by utilization efficiency of N. We conclude that future research should focus on optimum sweet potato cultivation technologies at Northern latitudes.

## Introduction

Global warming in hotter climates creates stressful conditions for plants, leading to reduced plant growth, shortened growth cycles and, in the case of tuber crops, delayed tuber formation [1,2]. Thus, the impact of climate on plants has been widely discussed in a negative context [3]. In recent years, however, sweet potato (*Ipomoea batatas* (L.) Lam) has been successfully cultivated in Central Europe as well as in the Nordic countries. Climate change might therefore allow cultivation of warm-climate crops in cooler climates [4–7]. Traditionally, the sweet potato as a subtropical crop has been grown in Africa, Asia, Oceania, Americas and in the subtropical and Mediterranean Southern Europe, including Spain and Portugal with recent expansion to Central European countries Germany and Poland [8–14].

Broad cultivation range of sweet potato indicates its high environmental adaptability. As crops in general, sweet potato has a high photosynthetic capacity that can be partly maintained even under suboptimal conditions due to the adaptive variability in leaf economic traits [15,16]. Nitrogen is often the main limiting nutrient in agricultural soils, and thus, adding N to plants increases their growth and productivity, but only until the optimum amount of N has been reached; this varies from crop to crop [17–19]. For example, in a field study of potato (*Solanum tuberosum* L.) throughout the growing period, N0 plants showed the lowest net assimilation rate (*A*) values, while the plants at the N treatment of 300 kg ha^-1^ showed the highest *A* values. However, in contrast to a monotonous positive relationship between N and *A*, the highest dry biomass of leaves, shoots, roots or tubers was achieved in the N treatment of 200 kg N ha^-1^ [18]. In contrast to potato, the N fertilization requirement was less for sweet potato, and 100 kg ha^-1^ of N was already sufficient for a high sweet potato tuber yield [17]. In fact, due to presence of endophytes, sweet potato can fix a certain amount of nitrogen (N) from the atmosphere, explaining the lower N requirement [20–22]. Over-fertilization reduced the amount and biomass of storage roots in sweet potato, while the above-ground dry biomass and leaf area continued to increase with increasing N dose [23], indicating that the N requirement for optimum yield is lower than that for optimum whole plant growth. In addition, tuber yields of sweet potato widely vary between cultivars, reflecting genetic differences as well as different N fertilization and growing season requirements [24]. In subtropical sweet potato cultivation areas, a 130-day cultivation period is needed for growing a high marketable yield [25]. However, in cooler climates, overall lower temperature and shorter growing season length might limit the uptake and use of N added to the plants (N utilization efficiency) and N utilization efficiency might also vary among the cultivars, potentially limiting the profitability of sweet potato cultivation at its Northernmost margin. So far, little information is available on the variation in growing season requirements for sweet potato cultivars promising for cool climates and on the interaction of growing season requirements with N fertilization in cool climates [26].

The main objectives of the current study were to characterize the optimal N-fertilizer dose and growing season length for the purple sweet potato cultivar focusing on biomass accumulation, photosynthetic characteristics and contents of C and N in plant organs. The relationship between the C content in leaves and dry weight content per tuber dry mass provides essential information on the nutritional value of tubers in relation to fertilization and the length of growing period of the plants. We hypothesised that 1) during a longer growing period sweet potato plants require more a higher amount of N, and achieve a greater yield, indicating an overall enhanced N utilization efficiency; 2) higher N application dose and longer growth period increase the C and N content in the above-ground shoots, roots and tubers, 3) higher N availability increases the net assimilation rate (*A*) and intrinsic water use efficiency (iWUE).

## Materials and methods

### Preparation of sweet potato planting material

Sweet potato cultivar ‘Purple Bud’ (purple peel and flesh) imported from the People’s Republic of China was selected for the trial. Production of shoots from tubers took place in boxes filled with commercial garden soil Biolan (Biolan Oy, Eura, Finland) that included slow release fertilizer (N:P:K ratio of 12 − 14 − 24) and Mg-containing limestone powder (4 kg m^-3^, soil pH =  6.0), under the following conditions: air temperature of 22 °C, air humidity of 65% and light intensity of 700 µmol m^-2^ s^-1^ (12-h day). Depending on the date the tubers were placed in the soil, the shoots grew for 40 to 45 days. At the time of planting, the shoots were 30 to 40 cm long, with no age-related differences.

### Experimental site

**Fig 1:**
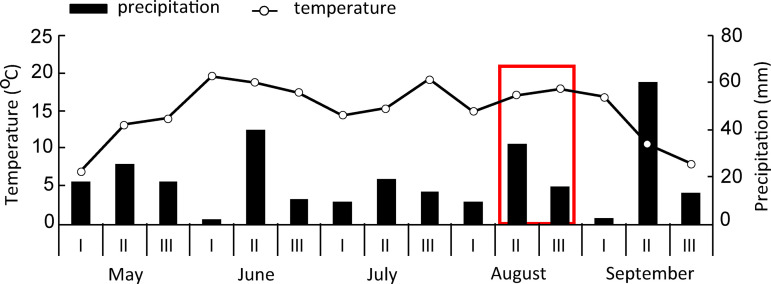
Ten-day average temperatures and amount of precipitation measured at the Eerika weather station in 2019. The red box indicates the period during which plant photosynthetic measurements were conducted.

The field sweet potato experiment was carried out in the Eerika field of the Rõhu experimental station of the Estonian University of Life Sciences (Chair of Crop Science and Plant Biology), Tartu County, Estonia (58°36′63.57ʺ N, 26°66′40.67ʺ E) in 2019. The preceding crops in the site were spring wheat (*Triticum aestivum* L. cv. ‘Hardena’) in 2017 and spring barley (*Hordeum vulgare* L. cv. ‘Anni’) in 2018. The soil of the experimental area was classified as a sandy loam Stagnic Luvisol [27]. The thickness of the plough layer was 27 − 30 cm [28], and the soil bulk density was 1.45–1.50 g cm^ −3^ [29]. In spring just before the planting, soil samples (*n* =  4) were collected at a depth of 20 cm, air-dried, and sieved (2 mm mesh size). Soil pH was determined in 1 M KCl solution (ratio 1:2.5) and soil organic carbon (*C*_org_) was quantified according to Tjurin method [30]. Total concentration of soil nitrogen (*N*_tot_) was measured with the Kjeldahl method [31]. At the beginning of the experiment, the humus horizon (plough layer) of the soil was characterized by the following indicators (mean ±  SE): pH_KCl_ =  6.03 ±  0.16, *C*_org_ 1.262 ±  0.048%, *N*_tot_ 0.109 ±  0.004%, plant-available P 71.9 ±  4.1 mg kg^-1^, K 128.1 ±  4.7 mg kg^-1^, Ca 1060 ±  26 mg kg^-1^, Mg 123.6 ±  3.2 mg kg^-1^. The weather data were collected from Eerika meteorological station (58^o^37' N, 26^o^66′ E), located about 2 km from the trial site (Fig 1) [7].

### Field study design

In the Northern Europe, the traditional planting time for frost-sensitive horticultural and field crops is the second week of June. In this study, planting of pre-grown shoots started on the 2nd of May and continued in 10-day intervals (10th of May, 20th of May, 30th of May, and 10th of June 2019). The trial was laid out in a randomized block design with four replications. In all treatments, the shoots were planted with a spacing of 40 cm. Each trial plot (2.1 m^2^) contained 7 plants.

The experiment involved four levels nitrogen (N) fertilizer YaraBela Axan NS 27-4 (Yara Estonia) (0 as control, 50, 100 and 150 kg N ha^-1^), 50 kg ha^-1^ was given on each planting date as starter fertilizer except the control plants. In addition, 50 kg ha^-1^ was given on the 18th of July (plants of 100 and 150 kg N ha^-1^ treatments), and 50 kg ha^-1^ on the 28th of July (plants of 150 kg N ha^-1^ treatment) (Fig 1). The fertilizer consisted of 13.5% of nitrate-N, 13.5% of ammoniacal-N, 4% of sulphur as CaSO_4_. Synthetic pesticides were not used. Weeds were mechanically removed four times during the growing season. After planting, the plants were watered twice with approximately 1 L of tap water per plant. In the first four planting treatments, plants were covered with a white cover veil of polypropylene (Baltic Agro, Estonia). The cover veil was removed after the danger of late frosts had passed. The tubers were harvested on the 26th of September after early night frosts a few days earlier.

### Gas exchange measurements

**Fig 2:**
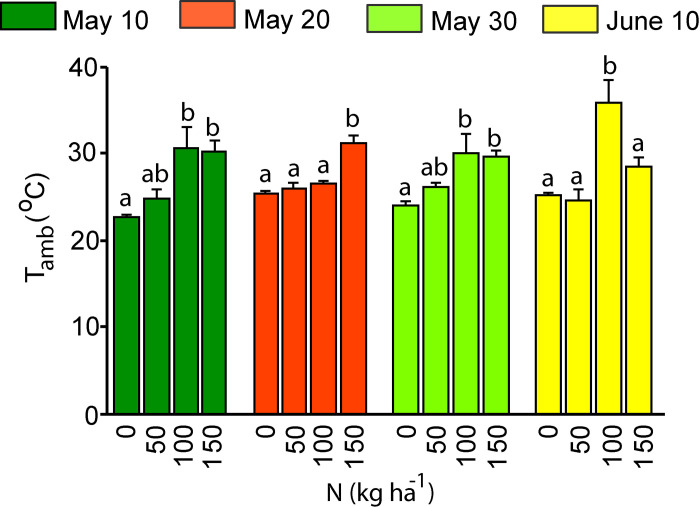
Ambient temperature (mean +  SE, n =  4) recorded by GFS-3000 gas-exchange system during photosynthesis measurements of sweet potato plants during the measurements (13-27 August, 2019). Planting date of sweet potato is shown as: dark green: the 10th of May, red: 20th of May, light green: 30th of May and yellow: 10th of June. The variation of ambient temperature within planting time was evaluated by one-way ANOVA. The ANOVA test was significant at *P* <  0.05, “ns” means a non-significant effect at *P* >  0.05.

Leaf photosynthetic measurements were conducted in the field from the 13th of August to the 27th of August. Fully-expanded mature leaves were selected for measurements, and the number of biological replicates (individual plants) was four for each treatment. A GFS-3000 portable gas exchange/fluorescence system that has a clip-on type leaf cuvette with an 8 cm^2^ window area (Heinz Walz GmbH, Effeltrich, Germany) was used. Ambient air passed through a dryer, CO_2_ absorber and humidifier at a flow rate of 750 μmol min^-1^. Chamber CO_2_ concentration was adjusted by the CO_2_ mixer and measurement light was provided by the leaf cuvette fluorimeter. After leaf insertion, the selected leaf was stabilized for 0.5 h under the CO_2_ concentration of 400 μmol mol^−1^, photosynthetically active quantum flux density of 1500 μmol m^−2^ s^−1^, cuvette block temperature of 25 °C and relative air humidity of 60% until steady-state gas-exchange rate was observed. After stomatal opening, net assimilation rate (*A*), stomatal conductance to water vapour (*g*_s_) and intercellular CO_2_ concentration (*C*_i_) were recorded. The ratio of *C*_i_ to ambient CO_2_ (*C*_a_) concentration (*C*_i_/*C*_a_) and intrinsic water use efficiency (iWUE =  *A*/*g*_s_) were calculated according to Flexas et al. [32]. In addition to the environmental characteristics measured at the Eerika weather station, the ambient temperature was also recorded in the field by the GFS-3000 gas-exchange system (Fig 2). The measurements started with N0-treated plants, followed by N100, N50 and finally N150-treated plants. In case of N0 and N50 plants, measurements started from the plants of the longest growing season and continued towards the shortest growing season. For N100 and N150 plants, measurements began from the plants of the shortest growing season and continued toward the longest growing season.

### Determination of carbon and nitrogen content, dry weight content and biomass

The plant material was harvested on the 25th of September by taking four plants from each trial plot to estimate the fresh mass of tubers, roots, leaves and stems directly after harvesting. The biomass fractions were further air-dried for 48 h at 80 °C (Sanyo Laboratory Convection Oven UFE 500), and their dry weight content (DW) was calculated. Before drying, the tubers and roots were cut into strips of ca. 2 cm. The contents of nitrogen (N) and carbon (C) of biomass fractions were determined by a Vario MAX CNS analyzer (Elementar Analysensysteme GmbH, Germany). The accuracy of leaf N content measurements was estimated to be 0.04% and that of C content estimates 0.5%.

### Statistical analyses

When needed, data were log-transformed. The effect of N fertilization, planting time and their interaction on C and N content in leaves and tubers (*C*_L_*, C*_T_*, N*_L_, *N*_T_), photosynthetic characteristics, yield of fresh above-ground shoots, roots and tubers (Shoot, Root, Tuber) and dry weight content in shoots, roots and tubers (*DW*_S_, *DW*_R_, *DW*_T_) was studied by two-way ANOVA. The variation of ambient temperature during photosynthesis measurements of plants and impact of N treatment on the above-mentioned characteristics within planting time were evaluated by one-way ANOVA. The relationships between *C*_T_ and *N*_T_, and *A* and *g*_s_ were evaluated by linear regressions. ANOVA tests and linear regression analyses were carried out using the OriginPro 2022 software (OriginLab Corporation, Northampton, USA). The differences were considered significant at *P* <  0.05.

## Results

### Weather conditions during the growing season and growing period length

In 2019, the growth period of sweet potato was dry and hot [7]. In May, the mean ( ± SE) air temperature was 11.4 ±  2.2 °C. There was a late night frost during the first ten-day period of May, and thus, the sweet potato planted on the 2nd of May, was killed (Fig 1). Mean air temperatures of 18.58 ±  0.63 °C and 16.26 ±  1.45 °C were recorded in June and July (Fig 1). In August, the temperature varied between 15 and 20 °C and by the end of September the temperature dropped below 10 °C (Fig 1) [7]. During the growing season, the total amount of precipitation was 285 mm, on average of 57 ±  13 mm per month and 1.90 ±  0.42 mm d^-1^. In the beginning of June and early September, the precipitation was 0.14 mm d^-1^ and 0.22 mm d^-1^, respectively. Most of the rain (34 to 60 mm) occurred in the middle of June, August and September. The growing period stopped prematurely due to early night frosts on the 23rd and 24th of September. Thus, the length of the growing period for the shoots of sweet potato planted on the 10th of May was 136 days, for shoots planted on the 20th of May 126 days, for shoots planted on the 30th of May 116 days and for shoots planted on the 10th of June 106 days.

### Impacts of N fertilization and planting time on the contents of N and C in leaves and tubers

**Fig 3:**
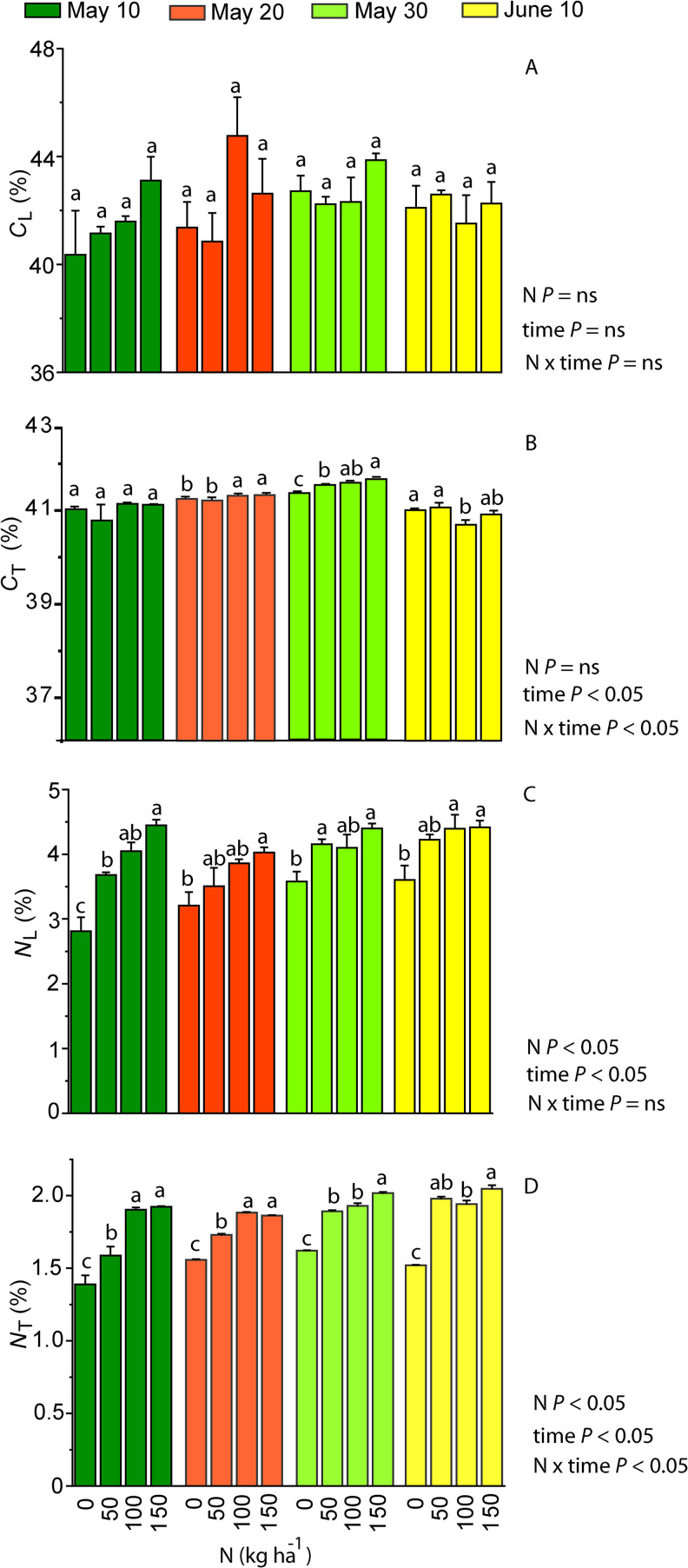
Mean values (n = 4, +  SE, %) of carbon (C) contents per leaf and tuber dry mass (*C*_L_, *C*_T_, A and B) and nitrogen (N) contents per leaf and tuber dry mass (*N*_T_, *N*_T_, C and D) in purple sweet potato cultivar in N0, N50, N100 and N150 treatments in different planting dates. Planting dates - dark green: the 10th of May, red: 20th of May, light green: 30th of May and yellow: 10th of June. The impact of N treatment, planting time and their interaction on C and N contents was evaluated with two-way ANOVA. Additionally, the impact of N treatment within each planting time was tested by a pairwise comparison t-test and is shown by the lowercase letters on top of the bars. The tests were significant at *P* <  0.05, “ns” means a non-significant effect at *P* >  0.05.

Carbon content per leaf dry mass (*C*_L_) remained stable at different nitrogen (N) application doses and planting dates, while carbon content per tuber dry mass (*C*_T_) was affected by the planting time of the plants as well as the interacting effect of N and time (Fig 3A and 3B). The lowest *C*_T_ values were found in plants from the longest growing period, while the highest *C*_T_ values in plants planted on the 30th of May (Fig 3B). *C*_T_ values increased along with the N application dose in plants planted on the 20th of May and 30th of May (Fig 3B).

**Fig 4:**
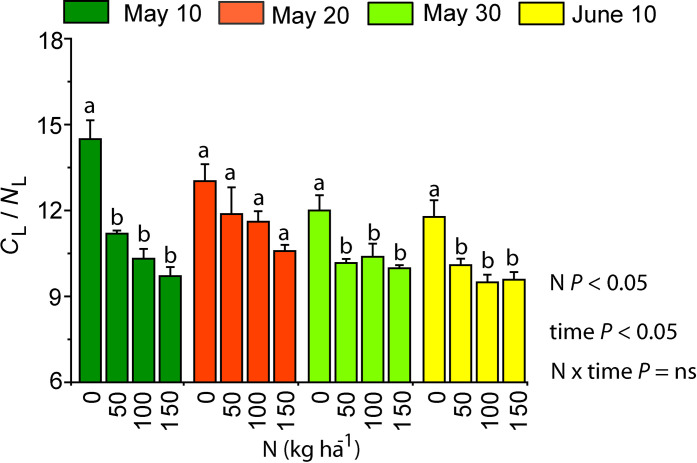
Ratio of carbon (C) and nitrogen (N) content (mean +  SE, n =  4) measured per leaf dry mass (*C*_L_/*N*_L_) in purple sweet potato cultivar in N0, N50, N100 and N150 treatments in different planting dates. Planting dates: the 10th of May (dark green), 20th of May (red), 30th of May (light green) and 10th of June (yellow). The impact of N, planting time and their interaction on C/N was evaluated with two-way ANOVA. The impact of N treatment within each planting time was tested by a pairwise comparison t-test and is shown by the lowercase letters on top of the bars. Both ANOVA tests were significant at *P* <  0.05, “ns” means a non-significant effect at *P* >  0.05.

**Fig 5:**
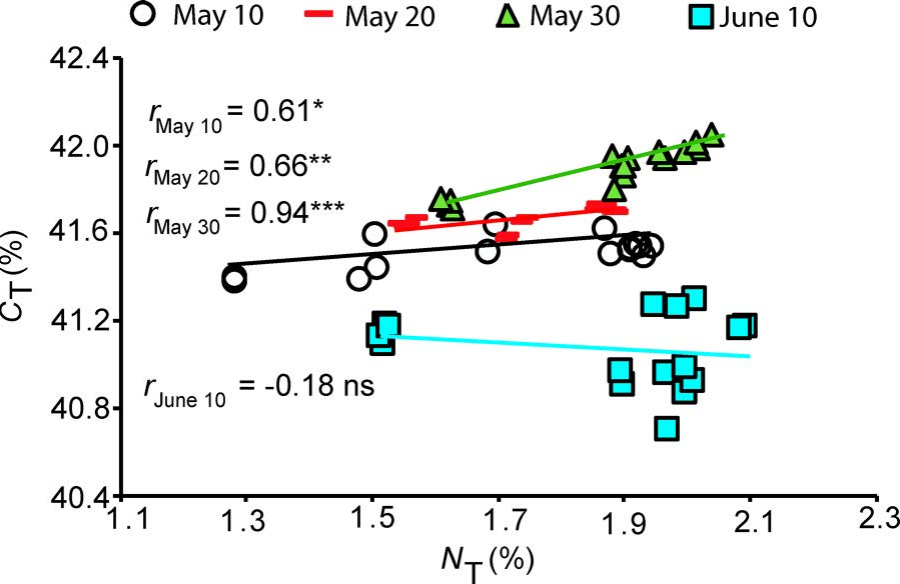
Relationships between carbon content per tuber dry mass (*C*_T_, %) and nitrogen content per tuber dry mass (*N*_T_, %) in purple sweet potato cultivar in N0, N50, N100 and N150 treatments in different planting dates. Different symbols show the planting time of the plants and each symbol contains plants of different N treatments: circle – the 10th of May (n =  16), dash – 20th of May (n =  16), triangle – 30th of May (n =  16), square – 10th of June (n =  16). Statistical significance of the relationships is shown as *P* <  0.05 (*), *P* <  0.05 (**) and *P* <  0.001 (***). “ns” means a non-significant relationship at *P* >  0.05.

The lowest N content per leaf dry mass (*N*_L_) and N content per tuber dry mass (*N*_T_) were observed in N0 plants, and the highest *N*_L_ and *N*_T_ values in N100 and N150 treatments (Fig 3C and 3D). *N*_L_ and *N*_T_ were the lowest in the sweet potato planted on the 10th of May and the highest in the plants planted on the 10th of June. *C*_L_/*N*_L_ ratio was the highest in the leaves and tubers of N0 plants (Fig 4). With increasing N application dose, also the *C*_T_*N*_T_ ratio showed a decreasing trend (data not shown). *C*_T_ and *N*_T_ were generally positively correlated, except for the plants planted in June (Fig 5).

### Photosynthetic characteristics of sweet potato in relation to N treatment

**Fig 6:**
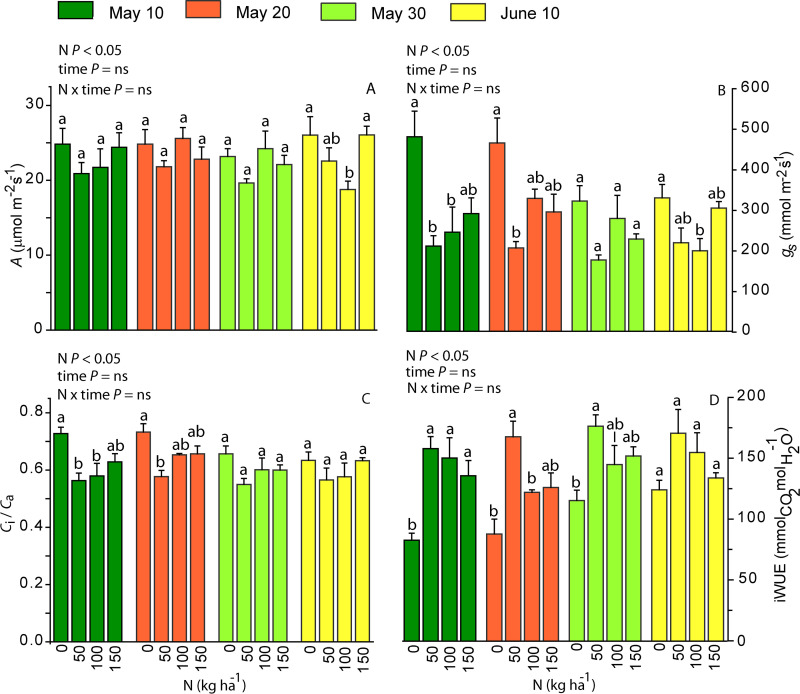
Mean values (n =  4, +  SE) of net assimilation rate (*A*, A), stomatal conductance (*g*_s_, B), ratio of intercellular CO_2_ to ambient CO_2_ (*C*_i_/*C*_a_, C) and intrinsic water use efficiency (iWUE, D) in sweet potato in N0, N50, N100 and N150 treatments in different planting dates. Planting dates: the 10th of May (dark green), 20th of May (red), 30th of May (light green) and 10th of June (yellow). The impact of N, planting time and their interacting effect on photosynthesis characteristics was evaluated with two-way ANOVA. The impact of N treatment within a planting time was tested by a pairwise comparison t-test and is shown by the lowercase letters on top of the bars. ANOVA tests were significant at *P* <  0.05, “ns” means a non-significant effect at *P* >  0.05.

**Fig 7:**
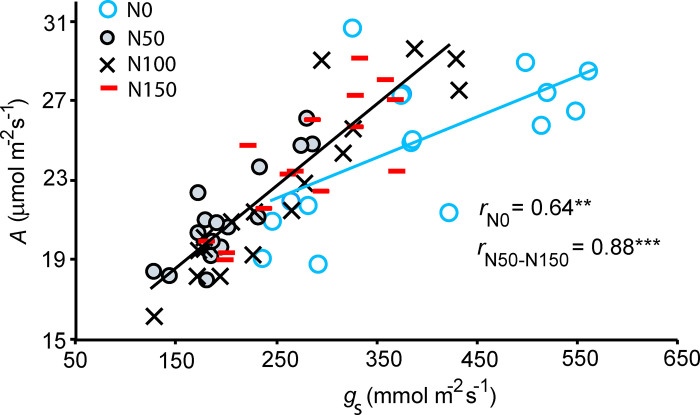
Relationships between net assimilation rate (*A*) and stomatal conductance (*g*_s_) in purple sweet potato cultivar in N0 (n = 16, blue circle), N50 (n =  16, grey circle), N100 (n =  16, black cross) and N150 (n =  16, red dash) treatments across all planting dates. The data were fitted by linear regressions and the statistical significance of the relationships is shown as *P* <  0.01 (**) and *P* <  0.001 (***).

Plant photosynthetic characteristics were only affected by the N application dose, whereas the changes did not strictly scale with N application dose. For example, the mean (±SE) net assimilation rate (*A*) of N0 plants (24.72 ±  0.91 μmol m^-2^ s^-1^) was significantly greater than that for N50 plants (23.84 ±  0.79 μmol m^-2^ s^-1^; Fig 6A). This was associated with the higher stomatal conductance (*g*_s_) in N0 plants (398 ±  29 mmol m^-2^ s^-1^) and lower *g*_s_ (202 ±  12 mmol m^-2^ s^-1^) in N50 plants (Fig 6B), leading to the higher ratio of *C*_i_ to ambient CO_2_ (*C*_a_) concentration (*C*_i_/*C*_a_) in N0 plants (0.688 ±  0.017) and lower *C*_i_/*C*_a_ ratio in N50 plants (0.564 ±  0.013; Fig 6C). Both, the *g*_s_ (262 ±  24 mmol m^-2^ s^-1^ for N100 and 279 ±  16 mmol m^-2^ s^-1^ for N150; Fig 6B) and *C*_i_*/C*_a_ (0.602 ±  0.019 for N100 and 0.629 ±  0.011 for N150; Fig 6C) were intermediate in N100 and N150 plants. As the result of similar *A* and large differences in *g*_s_, the intrinsic water use efficiency (iWUE = *A*/*g*_s_) was primarily driven by *g*_s_. iWUE was the lowest in N0 plants (Fig 6D). This was further supported by the relationships of *A* vs *g*_s_ that had a similar slope (equivalent to *A*/*g*_s_ ratio) for N50, N100 and N150 treatments, but *A* vs. *g*_s_ relationship levelled off at higher values of *g*_s_ (lower iWUE; Fig 7).

### Changes in shoots- and below-ground biomass and dry weight contents in dependence on N fertilization and date of planting

**Fig 8:**
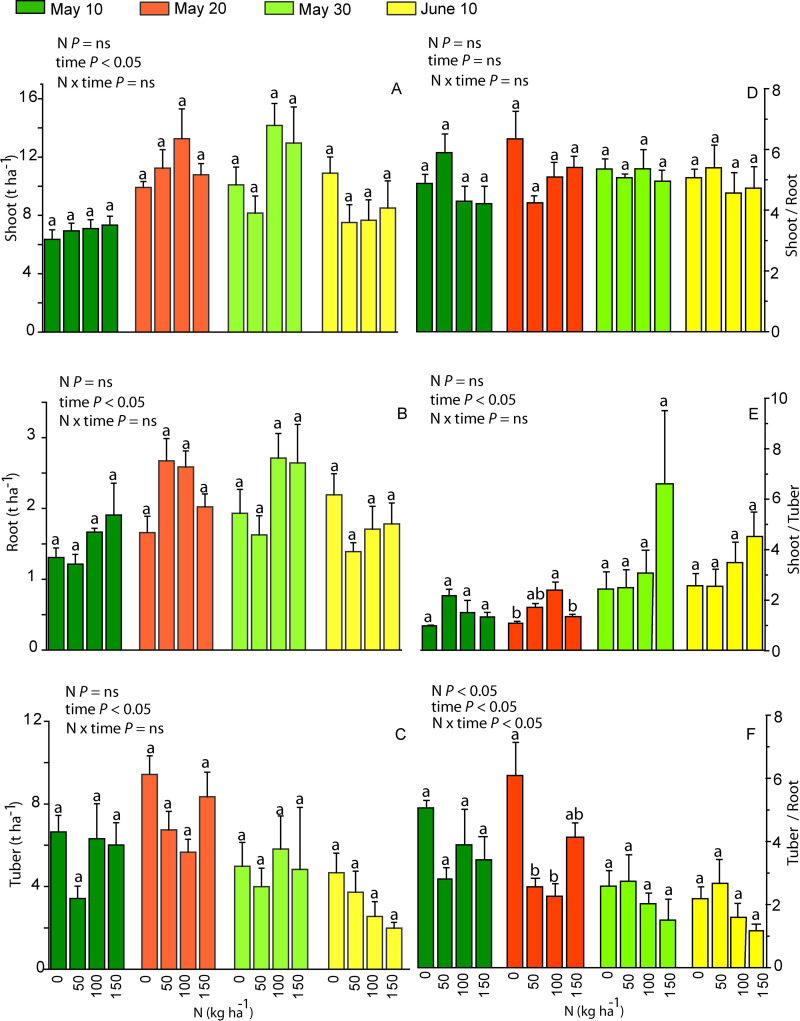
Mean values (n =  4, + SE) of fresh biomasses (t ha^-1^) of leaves and shoots (Shoot, A), roots (Root, B) and tubers (Tuber, C), and shoot to root fresh biomass ratio (Shoot/ Root, D), shoot to tuber fresh biomass ratio (Shoot/ Tuber, E) and tuber to root fresh biomass ratio (Tuber/ Root, F) in purple sweet potato cultivar in N0, N50, N100 and N150 treatments in different planting dates. Planting dates: the 10th of May (dark green), 20th of May (red), 30th of May (light green) and 10th of June (yellow). The impact of N, planting time and their interacting effect on yields and biomass ratios was evaluated with two-way ANOVA. In addition, the impact of N treatment within each planting time was tested by a pairwise comparison t-test and is shown by the lowercase letters on top of the bars. ANOVA tests were significant at *P* <  0.05, “ns” means a non-significant effect at *P* >  0.05.

Fresh shoot, root and tuber biomass depended only on the planting time (Fig 8A–8C). Sweet potato planted on the 20th of May had higher shoot and tuber biomass than the sweet potato planted on the 10th of May, the 30th of May and the 10th of June. There was no interacting effect between N dose and planting time on these traits (Fig 8A–8C). The ratio of the fresh shoot to root mass remained stable throughout the study (Fig 8D), while the ratio of the fresh shoot to tuber mass was higher for sweet potato plants planted on the 30th of May and the 10th of June (Fig 8E). Only the tuber to root mass ratio was affected by N treatment and planting time; it was the highest in the plants of N0 treatment and the ones planted on the 10th of May and the 20th of May (Fig 8F). The shoot to below-ground fresh mass ratio was higher in sweet potato planted in the end of May and the beginning of June than in sweet potato planted in the beginning of May (*P* <  0.05).

**Fig 9:**
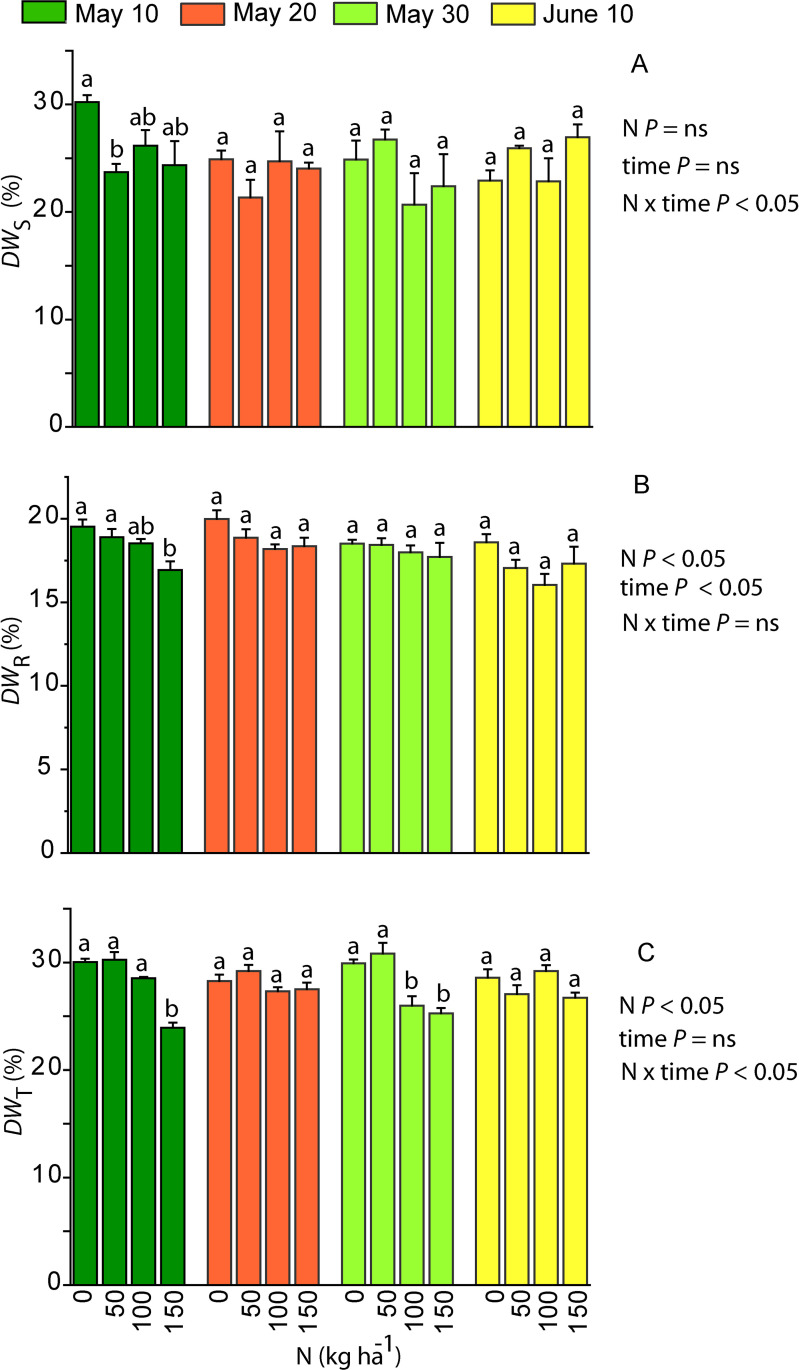
Mean values (n =  4, +  SE, %) of dry weight content in dry shoots (*DW*_S_, A), roots (*DW*_R_, B) and tubers (*DW*_T_, C) in purple sweet potato cultivar in N0, N50, N100 and N150 treatments and in different planting dates. Planting dates: the 10th of May (dark green), 20th of May (red), 30th of May (light green) and 10th of June (yellow). The impact of N, planting time and their interaction on DW was evaluated with two-way ANOVA. The impact of N treatment within each planting time was tested by a pairwise comparison t-test and is shown by the lowercase letters on top of the bars. ANOVA tests were significant at *P* <  0.05, “ns” indicates a non-significant effect at *P* >  0.05.

The dry weight content of shoots and leaves (*DW*_S_) remained unchanged in different N treatments (Fig 9A). However, the dry weight content of roots (*DW*_R_) decreased with increasing N application dose and shortening of the growing period (Fig 9B). Thus, the highest *DW*_R_ was observed in plants from N0 and N50 treatments and in plants planted on the 10th and the 20th of May. Similar effect of N treatment was found for *DW*_T_ (Fig 9C).

## Discussion

Due to climate change, Nordic summers are becoming increasingly hotter and drier. Hot weather causes stress to traditional local crops, resulting in reduced plant growth rates and a decline in crop yield quality and quantity [33]. However, climate change offers opportunities for experimenting with novel promising crop species such as sweet potato [7].

World population growth keeps putting pressure on agriculture as more food needs to be produced either by increasing yield per unit crop growth area or/and expansion of agricultural land area. Enhancement of yield per unit cultivation is frequently achieved by intensive agricultural practices, including fertilization, but such intensification can have major negative impacts on environment, e.g., due to nitrogen leaching to surface and ground water [34]. Sustainability of fertilization practices can be assessed by nitrogen utilization efficiency (NUE), defined as a ratio between the amount of N taken up and retained and the amount of N available to the crop [35]. NUE varies among different crops and plant genotypes and it also depends on agricultural practices [36–41]. The effects of preceding crops, crop protection and soil fertility management on NUE has been widely studied [42,43]. For example, in a previous study, cultivation of green manure caused significant differences in accumulation of nutrients in sweet potato leaves compared to spontaneous weeds [43]. Depending on the sensitivity of plants to soil nutrient availability, significant differences in leaf chlorophyll and protein content and carbon-nitrogen balance is expected [44–46]. A key management factor with potential to alter NUE is the length of the growing season. Especially in the higher latitude limits of crops with higher growing season degree sum requirement, extension of the growing season is expected to result in a greater NUE. In our study we analyzed how sweet potato biomass, dry weight content and photosynthetic characteristics are affected by N fertilization dose and planting date.

### Sweet potato photosynthesis in relation to N treatment

As the major constituent element of proteins and chlorophyll, nitrogen (N) plays a key role in plant physiological and metabolic processes. In purple sweet potato cultivar, the leaf N content (*N*_L_) increased with increasing N application dose (Fig 3), but the net assimilation rate (*A*) was only moderately affected (Fig 6A). In fact, the strength of the relationships between *N*_L_ and *A* varies for different crops and for any given crop [38]. Variations in N vs. *A* relationship can result from differences in allocation of N between proteins involved in photosynthesis and other metabolic processes, differences in investments among proteins of photosynthetic machinery, anatomical differences across plants and growth environment [46,47]. For example, a greater investment of *N* in chlorophyll and pigment-binding protein complexes increases light harvesting, but does not necessarily enhance light-saturated photosynthesis rate [48]. In our study, it is plausible that one reason for limited N fertilization effect on *A* despite greater *N*_L_ is a reduction of the fraction of N invested in rate-limiting proteins of photosynthetic machinery [49].

Our data also demonstrate that another key factor responsible for limited variation in *A* across fertilization treatments was a greater stomatal limitation of photosynthesis in N50, N100 and N150 treatments compared with N0 treatment as evidenced by lower stomatal conductance (*g*_s_), and reduced ratio of intercellular CO_2_ to ambient CO_2_ (*C*_i_/*C*_a_) in plants in fertilized treatments compared with N0 treatment (Fig 6B and 6C). Lower *g*_s_ is typically associated with a lower leaf water availability due to limited soil water availability or greater transpiratory water loss. Fertilization typically increases whole plant leaf area [50] and this can result in greater whole plant water loss and greater water limitation at given soil water availability, ultimately reducing *g*_s_. In our study, we did not assess whole plant leaf area, but aboveground biomass did not depend on fertilization dose (Fig 8A). Nevertheless, leaf dry mass per unit area (LMA) often decreases with increasing N availability [51,52]. As whole plant leaf area is given as leaf biomass/LMA, increased whole plant leaf area at higher fertilization doses is plausible in our study.

Changes in stomatal characteristics could also directly impact *g*_s_ in different fertilization treatments. A significant increase in stomatal density and a reduction in stomatal size has been observed in plant leaves when N application dose increased [53,54]. In general, there is an inverse relationship between stomatal size and density, and the overall impact of these changes on *g*_s_ depends on the shape of this relationship [55]. Significant differences in stomatal dimensions exist between different sweet potato cultivars [56]. In this study, we did not look at stomatal dimensions, and we suggest that future studies are needed to gain an insight into the reasons for fertilization-dependent reductions in *g*_s_, including possible changes in stomatal dimensions and whole plant leaf area.

According to Adams et al. (2017) the positive effect of N application dose on intrinsic water use efficiency (iWUE =  *A*/*g*_s_) varies across crops and different cultivars of the given crop [38]. In sweet potato, presence of endophytes that fix N from atmosphere may explain the poor relationship between photosynthesis characteristics and gradually increasing soil N [18,57]. Nevertheless, there is a large variability in how water use efficiency responds to N supply in sweet potato cultivars [58]. In our study, a lower iWUE was characteristic to plants in N0 treatment and there was no gradual increase in iWUE with increasing N application dose (Fig 6E). In contrast, in an earlier study the maximum water use efficiency (WUE, calculated as the ratio of photosynthesis to transpiration rates) was achieved with the highest N-fertilizer dose in the sweet potato cultivar ‘Jewel’, while in the cultivar ‘Tanzania’, WUE began to decrease after reaching a maximum level at an intermediate N application dose [58]. We suggest that further research is needed to examine how iWUE scales with fertilization dose across sweet potato cultivars with different responses of key functional traits - leaf N content, N allocation to proteins limiting photosynthetic capacity, stomatal dimensions and whole plant leaf area – to nitrogen availability.

### Dependence of tuber yield on N application dose and growing period length

In sweet potato cultivation, Fernandes et al. (2018, 2020) advise a careful selection of an appropriate N application dose to achieve a maximum tuber yield with a minimum fertilizer cost [40,42]. Root elongation, branching and tuber formation depend on carbohydrate supply by photosynthesis, and accordingly on how N availability affects whole plant leaf area and photosynthetic capacity per unit leaf area [59,60]. Apart from photosynthesis, tuber yield is also dependent on how fertilization affects biomass allocation. The effect of N on the above-ground biomass to below-ground biomass ratio varies between plant species as both positive and negative effects of nitrate N on root to shoot ratio have been shown [61]. In sweet potato, overfertilization can stimulate excessive growth of shoots and fibrous roots, but low allocation of photosynthates in the tuberous roots. Too low N availability, on the other hand, limits the growth of lateral roots and above-ground biomass [62]. In sweet potato, the ratio of *C*_L_ to *N*_L_, *C*_T_ to *N*_T_ and dry weight content per root dry mass (*DW*_R_) were the highest in N0 plants from the longest growing period (Fig 9B), indicating a greater nitrogen use efficiency for carbon accumulation in this treatment. Longer growing periods also resulted in a higher tuber yield (Fig 8C). Generally, growing season of sweet potato is 120 to 150 days, although early cultivars can be ready for harvesting in as soon as 90 days [63,64]. In 2019, due to the night frost on the 26th of September, the sweet potato planted on the 10th of June grew 107 days, while the ones planted on the 10th of May grew 138 days. The yield was especially low for sweet potato planted in June (Fig 8C), and clearly, the shortest growing period was insufficient to produce the tuber yield comparable to the sweet potato planted in the beginning of May. Taken together, our results demonstrate that the northern limit of commercial cultivation of sweet potato is determined by the minimum growing season length.

## Conclusions

We investigated the effects of N fertilizer dose and planting time on the physiological traits and yield of the purple sweet potato cultivar. The results demonstrated that N dose increased the C and N content per leaf dry mass that in turn affected the C and N contents per tuber dry mass. The N0 plants had the highest CO_2_ assimilation rate (*A*) and stomatal conductance (*g*_s_), while the N50 plants showed the lowest *A* and *g*_s_. Thus, the photosynthetic N use efficiency was curbed by limited stomatal conductance in N fertilization treatments, possibly reflecting greater transpiratory water loss due to a greater leaf area in fertilization treatments. Growing season length was the key factor affecting the tuber yield, and surprisingly no interactive effect between fertilization level and tuber yield was observed. Future studies are needed to investigate what plant structural, physiological and allocational traits limit the nitrogen utilization efficiency (biomass production per unit applied N) in sweet potato at its northernmost margin of cultivation.

Our study emphasizes that sweet potato is a promising crop for Nordic countries, but even at Northern latitudes, as it turns out, photosynthesis is severely limited by stomatal conductance. Thus, increasingly hotter and drier weather can limit the maximum yields of sweet potato in intensive agriculture with high N inputs. Thus, we argue that sweet potato cultivation at Northern latitudes requires breeding for varieties with enhanced water use efficiency.

## References

[1] Dahal K, Li X-Q, Tai H, Creelman A, Bizimungu B. Improving potato stress tolerance and tuber yield under a climate change scenario - A Current Overview. Front Plant Sci. 2019;10:563. doi: 10.3389/fpls.2019.00563 31139199 PMC6527881

[2] George TS, Taylor MA, Dodd IC, White PJ. Climate change and consequences for potato production: a review of tolerance to emerging abiotic stress. Potato Res. 2017;60(3–4):239–68. doi: 10.1007/s11540-018-9366-3

[3] Chaudhry S, Sidhu GPS. Climate change regulated abiotic stress mechanisms in plants: a comprehensive review. Plant Cell Rep. 2022;41(1):1–31. doi: 10.1007/s00299-021-02759-5 34351488

[4] Krochmal-Marczak B, Cebulak T, Kapusta I, Oszmiański J, Kaszuba J, Žurek N. The content of phenolic acids and flavonols in the leaves of nine varieties of sweet potatoes (*Ipomoea batatas* L.) depending on their development, grown in central Europe. Molecules. 2020;25(15):3473. doi: 10.3390/molecules25153473 32751600 PMC7436171

[5] Šlosár M, Hegedüsová A, Hegedüs O, Mezeyová I, Timoracká M. The effect of cultivar on selected quantitative and qualitative parameters of sweet potatoes (*Ipomoea batatas* L.) grown in Slovak republic. J Cent Eur Agric. 2020;21(2):344–53. doi: 10.5513/JCEA01/21.2.2684

[6] Kiemo FW, Tóth Z, Salamon P, Szabó Z. First report of sweet potato chlorotic stunt virus infecting sweet potatoes in Hungary. Plant Dis. 2021; 106(2):773. doi: 10.1094/PDIS-05-21-0944-PDN 34433315

[7] Kännaste A, Zekker I, Tosens T, Kübarsepp L, Runno-Paurson E, Niinemets Ü. Productivity of the warm-climate crop sweet potato (*Ipomoea batatas* L.) in northern latitudes. Zemdirbyste. 2023;110(4):311-318.

[8] Dawit M, Habte A. Yield and profitability of sweet potato (*Ipomoea batatas* (L.) Lam) as a function of increasing levels of phosphorus and varieties in southern Ethiopia. Appl Environ Soil Sci. 2023:2716227. doi: 10.1155/2023/2716227

[9] Drapal M, Rossel G, Heider B, Fraser PD. Metabolic diversity in sweet potato (*Ipomoea batatas*, Lam.) leaves and storage roots. Hortic Res. 2019;6:2. doi: 10.1038/s41438-018-0075-5 30603089 PMC6312539

[10] Ribeiro NP, Fernandes AM, Silva RM da, Pelvine RA, Assunção NS. Growth and yield of sweet potato in response to the application of nitrogen rates and paclobutrazol. Bragantia. 2021;80:e3821. doi: 10.1590/1678-4499.20200447

[11] Fiallo-Olivé E, García-Merenciano AC, Navas-Castillo J. Sweet potato symptomless virus 1: first detection in Europe and generation of an infectious clone. Microorganisms. 2022;10(9):1736. doi: 10.3390/microorganisms10091736 36144338 PMC9504438

[12] Maleita CMN, Santos DAF, Abrantes IM de O, Esteves I. First report of root knot nematodes *Meloidogyne incognita* and *M. javanica* parasitizing sweet potato, *Ipomoea batatas* L., in Portugal. Plant Dis. 2022. doi: 10.1094/PDIS-12-21-2680-PDN 35259309

[13] Tang W, Zhang Y, Liu Y, Wang X, Kou M, Yan H, et al. Quantifying cultivation technique and growth dynamics of purple-fleshed sweetpotato (*Ipomoea batatas* L.) in China. Field Crops Research. 2018;227:41–8. doi: 10.1016/j.fcr.2018.08.007

[14] Song W, Li C, Kou M, Li C, Gao G, Cai T, et al. Different regions and environments have critical roles on yield, main quality and industrialization of an industrial purple-fleshed sweetpotato (*Ipomoea batatas* L. (Lam.)) “Xuzishu8”. Heliyon. 2024;10(4):e25328. doi: 10.1016/j.heliyon.2024.e25328 38390079 PMC10881541

[15] Liu W, Zheng L, Qi D. Variation in leaf traits at different altitudes reflects the adaptive strategy of plants to environmental changes. Ecol Evol. 2020;10(15):8166–75. doi: 10.1002/ece3.6519 32788969 PMC7417217

[16] Luo XZ, Keenan TF, Chen JM, Croft H, Colin Prentice IC, Smith NG, et al. Global variation in the fraction of leaf nitrogen allocated to photosynthesis. Nat Commun. 2021;12(1):4866. doi: 10.1038/s41467-021-25163-9 34381045 PMC8358060

[17] Hartemink AE, Johnston M, O’Sullivan JN, Poloma S. Nitrogen use efficiency of taro and sweet potato in the humid lowlands of Papua New Guinea. Agric Ecosyst & Environ. 2000;79(2-3): 271-280. doi: 10.1016/S0167-8809(00)00138-9

[18] Salas-Rosales JE, Villa PM, Rodrigues AC, Rada F. Effects of nitrogen fertilization on the photosynthesis and biomass distribution in a potato crop. Peruv J Agron. 2020;4(2):68–74. doi: 10.21704/pja.v4i2.1571

[19] Dong HT, Li Y, Henderson C, Brown P, Xu C-Y. Optimum nitrogen application promotes sweetpotato storage root initiation. Horticulturae. 2022;8(8):710. doi: 10.3390/horticulturae8080710

[20] Terakado-Tonooka J, Fujihara S, Ohwaki Y. Possible contribution of Bradyrhizobium on nitrogen fixation in sweet potatoes. Plant Soil. 2013;367(1–2):639–50. doi: 10.1007/s11104-012-1495-x

[21] Yonebayashi K, Katsumi N, Nishi T, Okazaki M. Activation of nitrogen-fixing endophytes is associated with the tuber growth of sweet potato. Mass Spectrom (Tokyo). 2014;3(1):A0032. doi: 10.5702/massspectrometry.A0032 26819874 PMC4306741

[22] Döbereiner J, Reis VM, Paula MA, Olivares F. Endophytic diazotrophs in sugar-cane, cereals and tuber plants. In: Palacios R, Mora J, Newton WE, editors. New horizons in nitrogen fixation. Springer, Dordrecht; 1993. pp. 671-676. doi: 10.1007/978-94-017-2416-6_55

[23] Taranet P, Harper S, Kirchhof G, Fujinuma R, Menzies N. Growth and yield response of glasshouse- and field-grown sweetpotato to nitrogen supply. Nutr Cycl Agroecosyst. 2017;108(3):309–21. doi: 10.1007/s10705-017-9858-6

[24] Barkley SL, Schultheis JR, Chaudhari S, Johanningsmeier SD, Jennings KM, Truong V-D, et al. Yield and consumer acceptability of ‘Evangeline’ sweetpotato for production in North Carolina. hortte. 2017;27(2):281–90. doi: 10.21273/horttech03533-16

[25] Tipu MMH, Jahan R, Rahman J, Rahman MM, Islam MA, Apu MMRB. Development of efficient integrated management package against sweet potato weevil (*Cylas formicarius* [Fabricius, 1798]). Acta Agric Slov. 2021;117(4):1–4. doi: 10.14720/aas.2021.117.4.2066

[26] Wees D, Seguin P, Boisclair J. Sweet potato production in a short-season area utilizing black plastic mulch: effects of cultivar, in-row plant spacing, and harvest date on yield parameters. Can J Plant Sci. 2016;96 139–47. doi: 10.1139/cjps-2015-0150

[27] WRB. World Reference Base for Soil Resources. ISRIC; 2022. Available from: https://www.isric.org/sites/default/files/WRB_fourth_edition_2022-12-18.pdf.

[28] Reintam E, Köster T. The role of chemical indicators to correlate some Estonian soils with WRB and Soil taxonomy criteria. Geoderma. 2006;136(1–2):199–209. doi: 10.1016/j.geoderma.2006.03.028

[29] Madsen H, Talgre L, Eremeev V, Alaru M, Kauer K, Luik A. Do green manures as winter cover crops impact the weediness and crop yield in an organic crop rotation?. Biol Agric Hortic. 2016;32(3):182–91. doi: 10.1080/01448765.2016.1138141

[30] Burt R. Soil Survey Laboratory Methods Manual. 1st ed. Scientific Publishers; 2021.

[31] Van Reeuwijk LP. Procedures for Soil Analysis. 5th ed. International Soil Reference and Information Centre; 1995.

[32] Flexas J, Niinemets Ü, Gallé A, Barbour MM, Centritto M, Diaz-Espejo A, et al. Diffusional conductances to CO_2_ as a target for increasing photosynthesis and photosynthetic water-use efficiency. Photosynth Res. 2013;117(1–3):45–59. doi: 10.1007/s11120-013-9844-z 23670217

[33] Aubert L, Quinet M. Comparison of heat and drought stress responses among twelve tartary buckwheat (*Fagopyrum tataricum*) varieties. Plants (Basel). 2022;11(11):1517. doi: 10.3390/plants11111517 35684290 PMC9183088

[34] Abbasi MR, Sepaskhah AR. Nitrogen leaching and groundwater N contamination risk in saffron/wheat intercropping under different irrigation and soil fertilizers regimes. Sci Rep. 2023;13(1):6587. doi: 10.1038/s41598-023-33817-5 37085620 PMC10121562

[35] Prabhu G, Muthusamy SK, Bagavathiannan M, Mowrer J, Jagannadham PTK, Maity A, et al. Nitrogen use efficiency-a key to enhance crop productivity under a changing climate. Front Plant Sci. 2023;14:1121073. doi: 10.3389/fpls.2023.1121073 37143873 PMC10151540

[36] Wu Z, Luo J, Han Y, Hua Y, Guan C, Zhang Z. Low nitrogen enhances nitrogen use efficiency by triggering NO_3_- uptake and its long-distance translocation. J Agric Food Chem. 2019;67(24):6736–47. doi: 10.1021/acs.jafc.9b02491 31184154

[37] Kakabouki I, Togias T, Folina AE, Karydogianni S, Zisi C, Bilalis D. Evaluation of yield and nitrogen utilisation with urease and nitrification inhibitors in sweet potato crop (*Ipomoea batatas* L.). Folia Hortic. 2020;32(2):147–57. doi: 10.2478/fhort-2020-0014

[38] Adams MA, Buckley TN, Salter WT, Buchmann N, Blessing CH, Turnbull TL. Contrasting responses of crop legumes and cereals to nitrogen availability. New Phytol. 2018;217(4):1475–83. doi: 10.1111/nph.14918 29178286

[39] Ankumah RO, Khan V, Mwamba K, Kpomblekou A K. The influence of source and timing of nitrogen fertilizers on yield and nitrogen use efficiency of four sweet potato cultivars. Agric Ecosyst Environ. 2003;100(2–3):201–7. doi: 10.1016/s0167-8809(03)00196-8

[40] Fernandes AM, Campos LG, Senna MS, da Silva CL, Assunção NS. Yield and nitrogen use efficiency of sweet potato in response to cover crop and nitrogen management. Agron J. 2018;110(5):2004–15. doi: 10.2134/agronj2017.12.0721

[41] Swain EY, Rempelos L, Orr CH, Hall G, Chapman R, Almadni M, et al. Optimizing nitrogen use efficiency in wheat and potatoes: interactions between genotypes and agronomic practices. Euphytica. 2014;199(1–2):119–36. doi: 10.1007/s10681-014-1181-6

[42] Fernandes AM, Assunção NS, Ribeiro NP, Gazola B, da Silva RM. Nutrient uptake and removal by sweet potato fertilized with green manure and nitrogen on sandy soil. Rev Bras Ciênc Solo. 2020;44: e0190127. doi: 10.36783/18069657rbcs20190127

[43] Liu J, Xia H, Gao Y, Pan D, Sun J, Liu M, et al. Potassium deficiency causes more nitrate nitrogen to be stored in leaves for low-K sensitive sweet potato genotypes. Front Plant Sci. 2022;13:1069181. doi: 10.3389/fpls.2022.1069181 36561445 PMC9764221

[44] Hu W, Zhao W, Yang J, Oosterhuis DM, Loka DA, Zhou Z. Relationship between potassium fertilization and nitrogen metabolism in the leaf subtending the cotton (*Gossypium hirsutum* L.) boll during the boll development stage. Plant Physiol Biochem. 2016;101:113–23. doi: 10.1016/j.plaphy.2016.01.019 26874296

[45] Hu W, Coomer TD, Loka DA, Oosterhuis DM, Zhou Z. Potassium deficiency affects the carbon-nitrogen balance in cotton leaves. Plant Physiol Biochem. 2017;115:408–17. doi: 10.1016/j.plaphy.2017.04.005 28441628

[46] Evans JR, Clarke VC. The nitrogen cost of photosynthesis. J Exp Bot. 2019;70(1):7–15. doi: 10.1093/jxb/ery366 30357381

[47] Fathi A. Role of nitrogen (N) in plant growth, photosynthesis pigments, and N use efficiency: A review. Agrisost. 2022;28(1):1–8. doi: 10.5281/zenodo.7143588

[48] Kattge J, Díaz S, Lavorel S, Prentice IC, Leadley P, Bönisch G, et al. TRY – a global database of plant traits. Glob Change Biol. 2011;17(9):2905-2935. doi: 10.1111/j.1365-2486.2011.02451.x

[49] Niinemets Ü. Variation in leaf photosynthetic capacity within plant canopies: optimization, structural, and physiological constraints and inefficiencies. Photosynth Res. 2023;158(2):131–49. doi: 10.1007/s11120-023-01043-9 37615905

[50] Lieffering M, Andrews M, McKenzie BA. Effects of nitrogen on leaf growth of temperate cereals: A review. Agron Soc NZ. 1993;23:21–30.

[51] Khan A, Sun J, Zarif N, Khan K, Jamil MA, Yang L, et al. Effects of Increased N deposition on leaf functional traits of four contrasting tree species in Northeast China. Plants (Basel). 2020;9(9):1231. doi: 10.3390/plants9091231 32962033 PMC7570078

[52] Poorter H, Niinemets Ü, Poorter L, Wright IJ, Villar R. Causes and consequences of variation in leaf mass per area (LMA): a meta-analysis. New Phytol. 2009;182(3):565–88. doi: 10.1111/j.1469-8137.2009.02830.x 19434804

[53] Zhu K, Wang A, Wu J, Yuan F, Guan D, Jin C, et al. Effects of nitrogen additions on mesophyll and stomatal conductance in Manchurian ash and Mongolian oak. Sci Rep. 2020;10(1):10038. doi: 10.1038/s41598-020-66886-x 32572068 PMC7308411

[54] Juwarno S, Samiyarsih S. The effects of nitrogen fertilizer dosages on anatomical characteristics of *Ipomoea batatas* L. leaf. Biosfera. 2009;26(1):30–4.

[55] Haworth M, Marino G, Materassi A, Raschi A, Scutt CP, Centritto M. The functional significance of the stomatal size to density relationship: Interaction with atmospheric [CO_2_] and role in plant physiological behaviour. Sci Total Environ. 2023;863:160908. doi: 10.1016/j.scitotenv.2022.160908 36535478

[56] Samiyarsih S, Fitrianto N, Azizah E, Herawati W, Rochmatino. Anatomical profile and genetic variability of sweet potato (*Ipomoea batatas*) cultivars in Banyumas, Central Java, based on RAPD markers. Biodiversitas. 2020;21(4):1755-1766. doi: 10.13057/biodiv/d210460

[57] Yoneyama T, Terakado-Tonooka J, Minamisawa K. Exploration of bacterial N_2_-fixation systems in association with soil-grown sugarcane, sweet potato, and paddy rice: a review and synthesis. Soil Sci Plant Nutr. 2017;63(6):578–90. doi: 10.1080/00380768.2017.1407625

[58] Kelm M, Brück H, Hermann M, Sattelmacher B. Plant productivity and water use efficiency of sweet potato (*Ipomea batatas*) as affected by nitrogen supply. CIP Program Report 1999–2000. International Potato Center;2001:273–9.

[59] Salam BB, Barbier F, Danieli R, Teper-Bamnolker P, Ziv C, Spíchal L, et al. Sucrose promotes stem branching through cytokinin. Plant Physiol. 2021;185(4):1708–21. doi: 10.1093/plphys/kiab003 33793932 PMC8133652

[60] Freixes S, Thibaud M, Tardieu F, Muller B. Root elongation and branching is related to local hexose concentration in *Arabidopsis thaliana* seedlings. Plant Cell Environ. 2002;25(10):1357–66. doi: 10.1046/j.1365-3040.2002.00912.x

[61] Górska A, Lazor JW, Zwieniecka AK, Benway C, Zwieniecki MA. The capacity for nitrate regulation of root hydraulic properties correlates with species’ nitrate uptake rates. Plant Soil. 2010;337(1–2):447–55. doi: 10.1007/s11104-010-0540-x

[62] Omondi JO, Lazarovitch N, Rachmilevitch S, Yermiyahu U, Sperling O. High nitrogen availability limits photosynthesis and compromises carbohydrate allocation to storage in roots of *Manihot esculenta* Crantz. Front Plant Sci. 2019;10:1041. doi: 10.3389/fpls.2019.01041 31572405 PMC6749085

[63] Vimala B, Hariprakash B. Evaluation of some promising sweet potato clones for early maturity. Electron J Plant Breed. 2011;2(3):461–5.

[64] Ruangsuriya N, Sungthongwises K. Growth and yield response of sweet potato (*Ipomoea batatas* var. batatas) under acid sandy soil, northeast of Thailand. Agron Res. 2023;21(S3):1541–54. doi: 10.15159/AR.23.106

